# Clinical outcomes meta-analysis: measuring subendocardial perfusion and efficacy of transmyocardial laser revascularization with nuclear imaging

**DOI:** 10.1186/s13019-017-0602-8

**Published:** 2017-05-19

**Authors:** Jessika Iwanski, Shannon M. Knapp, Ryan Avery, Isabel Oliva, Raymond K. Wong, Raymond B. Runyan, Zain Khalpey

**Affiliations:** 1 Department of Medical Pharmacology, University of Arizona College of Medicine, Tucson, AZ USA; 2 Department of Cellular and Molecular Medicine, University of Arizona College of Medicine, Tucson, AZ USA; 3 Division of Cardiothoracic Surgery, Department of Surgery, University of Arizona, Tucson, AZ USA; 4 BIO5 Institute, Statistics Consulting Lab, University of Arizona, Tucson, AZ USA; 5 Department of Nuclear Medicine, Banner University Medical Center, Medical Imaging, Tucson, AZ USA; 6 Division of Cardiothoracic Surgery, Regenerative Medicine, Cellular & Molecular Medicine, University of Arizona College of Medicine, 1656 E. Mabel St, Rm 120, Medical Research Building, Tucson, AZ 85724 USA

**Keywords:** TMR, Angina, Coronary artery disease, Myocardial perfusion, Nuclear imaging, PET, SPECT, MUGA

## Abstract

**Introduction:**

Randomized and nonrandomized clinical trials have tried to assess whether or not TMR patients experience an increase in myocardial perfusion. However there have been inconsistencies reported in the literature due to the use of different nuclear imaging modalities to test this metric. The primary purpose of this meta-analysis was to determine whether SPECT, MUGA and PET scans demonstrate changes in myocardial perfusion between lased and non-lased subjects and whether laser type affects myocardial perfusion. The secondary purpose was to examine the overall effect of laser therapy on clinical outcomes including survival, hospital re-admission and angina reduction.

**Methods:**

Sixteen studies were included in the primary endpoint analysis after excluding all other non-imaging TMR papers. Standardized mean difference was used as the effect size for all quantitative outcomes and log odds ratio was used as the effect size for all binary outcomes.

**Results:**

Statistically significant improvements in myocardial perfusion were observed between control and treatment groups in myocardial perfusion at 6-month follow up using PET imaging with a porcine model. However non-significant differences were observed in patients at 3 and 12 months using SPECT, PET or MUGA scans. Both CO_2_ and Ho:YAG laser systems demonstrated an increase in myocardial perfusion however this effect was not statistically significant. In addition both laser types displayed statistically significant decreases in patient angina at 3, 6 and 12 months but non-significant increases in survival rates and decreases in hospital re-admissions.

**Conclusion:**

In order to properly assess myocardial perfusion in TMR subjects, subendocardial perfusion needs to be analyzed via nuclear imaging. PET scans can provide this level of sensitivity and should be utilized in future studies to monitor and detect perfusion changes in lased and non-lased subjects.

## Background

A significant number of patients currently suffering from coronary artery disease (CAD) experience severe ischemia due to multi-vessel atherosclerotic obstruction, leading to heart failure and impaired myocardial function [1]. Prophylaxis and treatment options for this population involves drug therapy, lifestyle changes, percutaneous coronary interventions (PCI) and coronary artery bypass grafting (CABG) [1, 2]. A large portion of these individuals suffer from refractory CAD not amenable to percutaneous or conventional surgical interventions [1–3]. For this patient population the extent of CAD is widespread and traditional revascularization alone is not sufficient to reinstate adequate flow through the coronary vessels. Transmyocardial revascularization (TMR) has emerged as an additional therapeutic option for these individuals. It has been reported to provide symptomatic angina relief with improved quality of life, decreased cardiac events and decreased cardiac re-hospitalizations [4–6]. More importantly, TMR has been utilized to treat patients who would normally not benefit from CABG procedures alone [4–6]. This surgical procedure can be performed as a stand-alone or hybrid therapy for severe patients who are not candidates for percutaneous interventions or who cannot be completely revascularized via CABG procedures [4, 5].

Numerous mechanisms have been proposed as the source of angina relief and improved cardiac function noticed in patients receiving TMR therapy [7]. Denervation [1, 6], angiogenesis [8–11], and redistribution of wall stress [12] have been attributed to the postoperative improvements noted in severe angina patients [13]. Despite the positive reverberations of laser therapy in these individuals, it still remains unclear whether TMR increases myocardial perfusion within an infarcted myocardium. Different imaging modalities have been employed to monitor patients postoperatively and to detect changes in the functional status of the heart. Reversible ischemia and regional myocardial wall motion have been assessed using stress testing with various contrast mediums via echocardiography (ECHO) [14, 15]. In addition, magnetic resonance imaging (MRI) and computed tomography (CT) have been utilized to determine changes in cardiac pathology and define the adequacy of perfusion established in ischemic or infarcted myocardial tissue [16]. Nuclear medicine has emerged as a minimally invasive method of objectively quantifying myocardial perfusion and viability post TMR treatment and revascularization. Nuclear stress tests such as myocardial perfusion scintigraphy (thallium 201 and technetium-99 m sestamibi (99 m-Tc)), single photon emission computed tomography (SPECT), multigated acquisition scan (MUGA), and positron emission tomography (PET) have been more commonly employed to measure the extent of ischemic burden and recovery in patients with ischemic cardiomyopathy [17]. Nuclear imaging has a reported higher sensitivity for measuring myocardial viability and for evaluating the clinical outcomes of revascularization in contrast to echocardiography which has a greater specificity for assessing contractility [18]. The intent of this meta- analysis was to evaluate the effect of transmyocardial laser revascularization on myocardial perfusion by analyzing results following nuclear imaging tests. Different modalities of nuclear imaging will be assessed and compared to determine if laser therapy can provide proper revascularization and adequate perfusion in patients with depressed ventricular function suffering from ischemic heart disease.

## Purpose

This meta-analysis examined the literature on sole and adjunctive TMR/CABG therapy in order to determine the effects of laser treatment on patients with ischemic cardiomyopathy. Specifically, this paper had two endpoints:

(a) Investigate the effect of laser treatment on myocardial perfusion using different nuclear imaging modalities under rest and stress conditions at short-term and long-term follow-up.
  (b) Compare the overall effects of CO_2_ and Ho:YAG laser systems on myocardial perfusion and ischemia and determine whether there is a significant difference in these outcomes between laser types.
Establish the effects of CO_2_ and Ho:YAG laser therapy on patient clinical outcomes including angina reduction, survival, and hospital re-admission.

## Methods

### Literature search

A comprehensive search was performed using the research engines PUBMED, ScienceDirect, and MEDLINE (via EBSCOHost and OvidSP). Keywords used to identify relevant studies were: “transmyocardial revascularization imaging, TMR and TMLR perfusion, TMLR imaging, TMR, TMR and TMLR angina, TMR and TMLR refractory angina, TMLR and TMR PET scans, nuclear imaging and TMR, TMR versus medical management, nuclear imaging TMR and CABG, myocardial perfusion, and TMR versus control”. Published articles were examined from the earliest date possible to the current date, January 2016. All numerical data was extracted directly from the study text and/or tables. If percentage values were given, only then, were calculations made in order to determine the exact number of patients in an outcome group. No assumptions were made from pictorials or graphs unless a precise *p*-value, mean ± SD or SEM was provided.

### Inclusion/exclusion criteria

For this meta-analysis, eligible studies had to be randomized or non-randomized trials that compared TMR treatment groups with control participants (TMR versus medical management, TMR versus sham, TMR/CABG versus CABG, or TMR/CABG versus TMR). Trials that examined pre versus post treatment data were excluded from the statistical analysis. Three papers (Hughes [19–21]) used porcine subjects randomized to either TMR treatment or sham thoracotomy. These were included in the meta-analysis in order to provide additional data regarding PET nuclear imaging.

Studies examining the effects of CO_2_ and/or Ho:YAG laser systems were included and papers using excimer laser treatments were excluded. All procedures were performed via a left thoracotomy or median sternotomy and none were executed via percutaneous methods.

Myocardial perfusion and ischemia were measured in subjects who had undergone nuclear rest and stress testing. For this analysis only PET, MUGA and SPECT scans were used to measure these parameters. MRI and ECHO imaging were excluded and may be considered in future analyses.

#### Definition of endpoints

The primary endpoint of this meta-analysis was to evaluate the effect of lasers on myocardial perfusion using different imaging modalities. Since various studies utilized different terms to describe perfusion effects in subjects with ischemic cardiomyopathy, myocardial perfusion was defined as the rate of blood flow or perfusate through the heart muscle. Under this condition, terms such as “peak filling rate, peak ejection rate, myocardial perfusion and perfusion defect” were included to denote perfusion through the heart. Likewise various studies used different terminology to describe ischemic areas or zones within the heart. In this meta-analysis studies using the terms hibernating, fibrotic, or reversible ischemia were classified together as reversible ischemia. Studies that did not specify whether a defect was reversible or irreversible were simply categorized as ischemic. All other continuous outcomes reported by imaging studies were recorded and analyzed. After assimilating terms there was a final list of five outcome measures included in this meta-analysis: LVEF, LVEDV, ischemia, reversible ischemia, and myocardial perfusion.

Nuclear imaging techniques were narrowed down to the three major types currently used for perfusion diagnostics: SPECT, MUGA and PET scans. Studies which quoted the use of MIBI, sestamibi, QGSPECT, or thallium 201 stress testing, were all categorized as SPECT imaging. In addition all types of intravenous contrast mediums and stressing agents were included in each imaging type.

The second purpose of this meta-analysis was to examine the clinical outcomes of laser therapy at short and long-term follow up. Survival, hospital re-admission and angina reduction were chosen as important clinical effects of laser treatment. In these studies hospital re-admissions were due to unstable angina and acute MI’s.

### Data extraction

Numerical data was extracted directly from eligible papers and recorded into a master file that was analyzed by a statistician. For quantitative outcomes, values collected included means, sample sizes, standard deviations, SEMs, and/or *p*-values for both treatment and control groups. For categorical outcomes, values extracted were total participants present at baseline and total participants present in a category at a given time point, for both treatment and control groups. Continuous and binary outcomes were independently recorded by two individuals and cross checked, to ensure no discrepancies arose between collected data.

### Data analysis

All analyses were conducted in Program R version 3.2.2 [22]. Standardized mean difference [23] was used as the effect size for all quantitative outcomes and log odds ratio was used as the effect size for all binary/categorical outcomes; in each case the escalc() function in the R package ‘metafor’ version 1.9.8 [24] was used except in cases where only the *p*-value was available, in which case the p_to_d2() function in the R package ‘MAd’ version 0.8.2 was used [25]. In order for results to have consistent interpretation, values were transformed so that a positive difference in means (quantitative outcomes) or log-odds ratios (binary/categorical outcomes) between TMR treatment and control would always indicate that TMR performed better than the control and a negative difference would always indicate TMR performed worse than the control. For analyses that included multiple outcomes or time points within a study, a correlation of 0.5 was assumed among outcomes within a study and the combined effect across outcomes or time points was computed using the agg() function in the R package ‘MAd’. To evaluate the sensitivity of this assumption, analyses were run with correlations of 0, 0.5 and 0.99 among outcomes within a study. Each correlation was checked to determine whether there were differences between different correlation conclusions. Since no differences were noticed, results shown are for analyses using a correlation of 0.5. Random effects models with the Knapp and Hartung [26] adjustment were fit using the rma() function in the R package ‘metafor’ version 1.9.8 [24].

## Results

### Search results

**Fig. 1:**
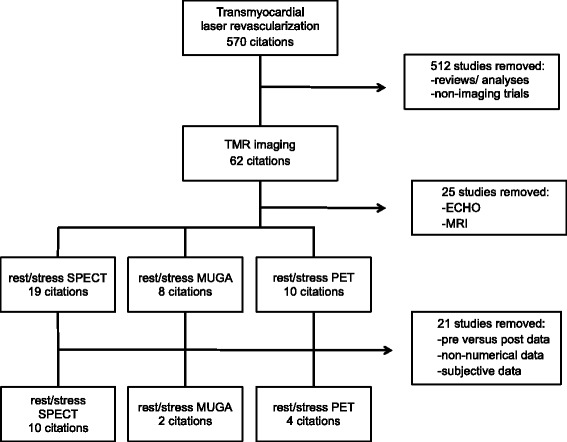
Criteria for eligible nuclear imaging studies. The selection process consisted of 62 citations selected from a total 570 TMLR published papers. From these imaging studies, ECHO and MRI trials were removed. Lastly the final 16 studies were screened for trials, which included objective data, represented by numerical values in graphs, tables or within the published text

Electronic databases yielded 570 TMLR citations, which were screened specifically for nuclear imaging studies, resulting in a total of 62 citations (Fig. 1). From these papers, 25 studies using ECHO and MRI imaging were removed. Papers that included more than one imaging modality (ie. ECHO and SPECT) remained in the selection pool, however values related only to the appropriate imaging type were recorded. The remaining 37 citations were further screened, and studies containing pre versus post treatment data, subjective data or non-numerical data, in which no true values were presented in the paper (ie. graphical data), were excluded. This resulted in a final group of 16 papers used in the primary endpoint analysis.

Tables 1 and 2 display the characteristics of studies used in the analysis for primary and secondary endpoints, respectively. A total of 1753 subjects were recorded at baseline (Table 1), 880 of which were designated into the control arm and 867 designated into the treatment group. Patients who began in the control group and later crossed over to the treatment group due to severe angina were included in the treatment analysis [27] and patients who received alternative treatments (ie. VEGF therapy) were excluded from the treatment arm [28]. Of the 16 papers discussed in the primary endpoints, 7 used CO_2_ lasers and 9 used Ho:YAG laser systems.

**Table 1:** Characteristics of studies used for primary endpoints

Study name	Groups	Sample size (n)	Laser type	Imaging	Duration (months)
Aaberge 2001 [27]	TMR vs MM	100	CO_2_	SPECT/MUGA	12
Burns 2001 [26]	TMR vs MM	188	CO_2_	SPECT	12
Frazier 1999 [29]	TMR vs MM	192	CO_2_	SPECT	12
Aaberge 2000 [11]	TMR vs MM	100	CO_2_	MUGA	12
Allen 1999 [33]	TMR vs MM	275	Ho:YAG	SPECT	12
Burkhoff 1999 [31]	TMR vs MM	182	Ho:YAG	SPECT	12
Hughes 1999 [21]^a^	TMR vs SHAM	10	Ho:YAG	PET	6
Hughes 2000a [22]^a^	TMR vs SHAM	10	CO_2_	PET	6
Hughes 2000b [22]^a^	TMR vs SHAM	10	Ho:YAG	PET	6
Tio 2004 [28]	TMR vs MM	25	Ho:YAG	PET	3
Leon 2005 [20]	TMR vs SHAM	200	Ho:YAG	SPECT	6
Diegeler 1998 [24]	TMR vs CABG/TMR	28	Ho:YAG	SPECT	3
Schneider 2001 [25]	TMR vs CABG/TMR	43	Ho:YAG	SPECT	12
Schofield 1999 [34]	TMR vs MM	188	CO_2_	SPECT	12
Hughes 2002 [23]^a^	TMR vs SHAM	10	Ho:YAG	PET	6
March 1999 [19]	TMR vs MM	192	CO_2_	SPECT	12

Sample sizes represented are at baseline

^a^Hughes papers ([21–23]) used a porcine model

*TMR* transmyocardial revascularization, *MM* medical management, *CABG* coronary artery bypass graft

**Table 2:** Characteristics of studies used for secondary endpoints

Study name	Groups	Sample size (n)	Laser type	Outcome measure
Frazier 1999 [29]	TMR vs MM	19	CO_2_	survival, angina reduction, re-admission
Frazier 2004 [49]	CABG vs TMR/CABG	44	CO_2_	survival, angina reduction
Allen 2000 [30]	CABG vs TMR/CABG	263	Ho:YAG	survival
Allen 2004 [51]	CABG vs TMR/CABG	218	Ho:YAG	survival
Aaberge 2000 [11]	TMR vs MM	100	CO_2_	survival, angina reduction, re-admission
Allen 1999 [33]	TMR vs MM	275	Ho:YAG	survival, angina reduction
Burkhoff 1999 [31]	TMR vs MM	182	Ho:YAG	survival, angina reduction, re-admission
Leon 2005 [20]	TMR vs SHAM	200	Ho:YAG	survival, angina reduction
Diegeler 1998 [24]	TMR vs TMR/CABG	40	Ho:YAG	survival
Schneider 2001 [25]	TMR vs TMR/CABG	68	Ho:YAG	angina reduction
Schofield 1999 [34]	TMR vs MM	188	CO_2_	survival, angina reduction

N numbers represented are at baseline of study

Angina reduction is a reduction of at least two or more CCS classes

*TMR* transmyocardial revascularization, *MM* medical management, *CABG* coronary artery bypass graft

Table 3 demonstrates the baseline characteristics of participants included in control (MM and CABG) and treatment (TMR and CABG/TMR) groups in this meta-analysis. Not all studies provided similar baseline information therefore a direct comparison of both groups from all papers was not possible. Of note, the Hughes papers (1999, 2000 and 2002) were excluded from this table since they utilized porcine models. From the remaining data, it was determined that there was no statistical significance between control and treatment arms in patient demographics and medical history. However statistically significant differences

**Table 3:** Baseline characteristics of clinical trials in primary and secondary endpoints

	Control (%)	Treatment (%)	*p* value	no. studies
Demographics				
Female	29.5	29.6	0.92	14
Mean age	62.1	61.8	0.32	13
Medical History				
CABG	66.7	66.9	0.37	14
PTCA	31.5	29.4	0.49	12
Acute MI	62.7	62.8	0.95	15
CHF	21.6	20.5	0.74	7
Cardiac Status				
Mean LVEF	49.4	49.5	0.95	11
NYHA Class III	55.6	52.6	0.19	8
NYHA Class IV	44.4	47.4	0.19	8
Unstable angina	9.0	24.2	<0.01	5
Risk Factors				
HTN	64.5	62.9	0.55	9
Hypercholesterolemia/HLD	80.6	73.2	0.01	5
DM	36.8	35.7	0.65	13
Tobacco use	31.4	30.6	0.56	11

control = MM and CABG treatment = TMR and CABG/TMR

were noticed in patients’ cardiac status and risk factors (unstable angina and hypercholesterolemia/HLD levels (*p* < 0.01 and *p* = 0.01, respectively). It is expected that study groups would differ in unstable angina as patients with severe angina are typically placed into TMR or CABG/TMR treatment arms. Participants with a worse prognostic outcome are preferentially placed into these therapeutic groups, as they are viewed to be more beneficial to this population. This becomes apparent when the proportions of unstable angina patients are further broken down between MM, TMR, CABG and TMR/CABG groups (6.66%, 21.6%, 52.4% and 65.2%, respectively). This is evidence of selection bias, which is present in non-randomized clinical trials. From the 16 papers included in the primary endpoints, 3 were excluded from this analysis as they used porcine subjects [19–21] Of the remaining 13 studies only two were non-randomized clinical trials [29, 30]. Therefore, the majority of eligible papers consisted of randomized clinical trials (RCT). In general, the baseline characteristics appeared to favour the control group as they had a significantly lower rate of unstable angina (15.2% decrease), despite higher rates of hypercholesterolemia/HLD (7.4% increase). Consequently the outcomes of this meta-analysis most likely yielded conservative results regarding the effect of adjunctive TMR therapy.

Study selection criteria was similar in all eligible studies. Many trials excluded patients who were > 75 years, had an LVEF <30%, advanced heart failure and inability to undergo study tests. Eligible patients had to have a history of coronary artery disease with refractory angina, classified as either CCS class III or CCS class IV, despite receiving optimal medical management on a maximal tolerable dose. For patients receiving sole TMR therapy, they had to present with areas of reversible ischemia, which were considered ineligible for percutaneous coronary interventions or surgical revascularization procedures. For patients undergoing CABG and/or CABG/TMR, they were required to have suitable vessels for grafting as well as demonstrable ischemic areas not amenable to direct revascularization by vein grafts. Patients who had a recent MI (within the last 6 months), chronic atrial fibrillation, myocardial wall thickness <9 mm (assessed by TTE) or major life-threatening comorbidities were excluded from study populations.

### Part I

#### Imaging analyses

**Fig. 2:**
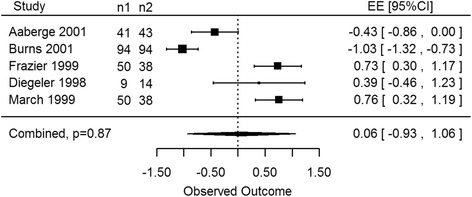
CO2 and Ho:YAG: stress SPECT at 3 months. Laser therapy showed an increase in myocardial perfusion however this effect was not statistically significant as compared to control groups (*p* = 0.87)

All imaging analyses included both CO_2_ and Ho:YAG laser systems as well as all continuous outcomes (LVEDV, LVEF, ischemia, reversible ischemia and myocardial perfusion) reported in studies using imaging techniques. For patients receiving TMR therapy there were no statistically significant differences noted in perfusion outcomes at rest measured via SPECT both at 3 and 12-month follow-ups (EE -2.68, 95% CI -14.05 to 8.67, *p* = 0.42, EE -0.18, 95% CI -0.74 to 0.37, *p* = 0.41, respectively). Even though the overall estimated effect (EE) favoured TMR at 3 months via SPECT imaging under stress, this was not considered statistically significant (EE 0.06, 95% CI -0.93 to 1.06, *p* = 0.87, Fig. 2). Likewise non-significant results were also measured at 12 months under stress conditions (EE -0.72, 95% CI -5.02 to 3.58, *p* = 0.63). This was most likely influenced by the results of Burns et al. [31], as they reported 188 patients in their study with no overall increase in myocardial perfusion in lased regions.

**Fig. 3:**
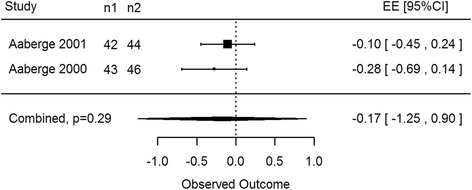
CO2 and Ho:YAG: rest MUGA at 12 months. No statistical significance was observed in myocardial perfusion between laser therapy and control participants via MUGA scans (*p* = 0.29)

MUGA scans were predominantly used to determine the LVEF of patients post treatment and were usually reported in addition to another imaging modality [32]. Results in this section included only two papers (Aaberge [13] and Aaberge [32]) comparing TMR with medical management, however both studies examined 180 participants collectively with MUGA scans. At rest there was no statistically significant difference between control and treatment groups at 3 months (EE -0.28, 95% CI -1.78 to 1.21, *p* = 0.25) and 12 months (EE -0.17, 95% CI -1.25 to 0.90, *p* = 0.29, Fig. 3). Only one paper displayed results for patients under stress conditions, Aaberge [32] Again no significant difference was found between groups at 3 months (EE -0.21, 95% CI -0.58 to 0.16, *p* = 0.27) and 12 months (EE -0.03, 95% CI -0.40 to 0.34, *p* = 0.88).

**Fig. 4:**
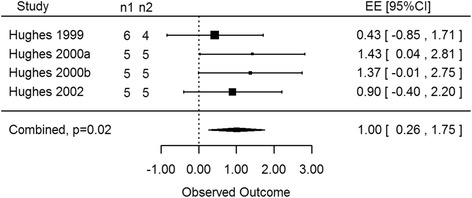
CO2 and Ho:YAG: rest PET at 6 months. Statistical significance was determined in myocardial perfusion between laser therapy and control groups (*p* = 0.02) using porcine subjects via PET scans

Considering the novelty and cost of utilizing PET scans for patient follow-up, only one RCT was found to report the effects of TMR via this imaging technique [33]. This study included 25 patients randomized to MM or TMR therapy. The three additional papers incorporated in this subsection used porcine subjects randomized to TMR therapy or sham thoracotomy [19–21]. Under resting conditions, PET scans showed increases in perfusion outcomes with TMR therapy versus control at 3 months (EE 0.25, 95% CI -0.53 to 1.04, *p* = 0.53) and 6 months (EE 1.00, 95% CI 0.26 to 1.75, *p* = 0.02, Fig. 4). Results were statistically significant at 6-month follow-up, however only included studies performed on porcine models. During stress conditions there was a non-significant difference between control and treatment groups at 3 months (EF -0.08, 95% CI -0.76 to 0.60, *p* = 0.81). There was insufficient data to complete analysis at 6 or 12 months post treatment during stress conditions.

#### Comparison of laser types

**Fig. 5:**
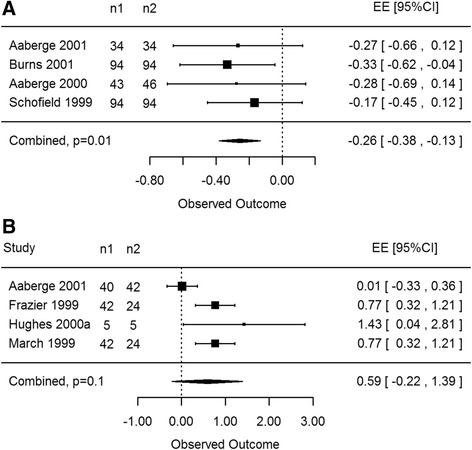
**a** CO2: LVEF at 12 months. A statistically significant decrease in LVEF was determined in laser therapy patients as compared to control patients (*p* = 0.01). **b** CO2: myocardial perfusion at 6 and 12 months. The CO_2_ laser showed an increase in myocardial perfusion compared to control groups, however this effect was not statistically significant (*p* = 0.10)

CO_2_ and Ho:YAG lasers were analyzed individually for their effect on LVEF, myocardial perfusion, and ischemia for up to 12 months of follow up. Studies that reported these outcome measures with SPECT, MUGA and/or PET under rest or stress conditions were included in the analysis. To determine if CO_2_ lasers have an effect on LVEF, studies using SPECT or MUGA were analyzed collectively under stress and rest conditions. At 12 months of follow-up, medical management groups showed a statistically significant improvement in LVEF compared to treatment groups (EE -0.26, 95% CI -0.38 to -0.13, *p* = 0.01, Fig. 5a). However CO_2_ lasers did not have a statistically significant effect on reversible ischemia under rest or stress SPECT scans compared to medical management groups (EE -0.50, 95% CI -2.76 to 1.75, *p* = 0.22). Although CO_2_ lasers showed an improvement in myocardial perfusion in the treatment group, this effect was not considered significant (EE 0.59, 95% CI -0.22 to 1.39, *p* = 0.10, Fig. 5b) up to and including 12 months of follow-up, as reported by MUGA, SPECT and PET scans collectively. Studies in this analysis included TMR versus MM groups, one of which was from a porcine model comparing TMR versus sham thoracotomy [20].

**Fig. 6:**
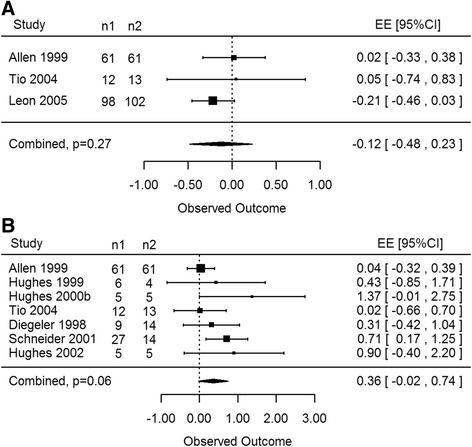
**a** Ho:YAG: ischemia up to 12 months. There was no statistical significance noted in ischemia between laser and control patients using the holmium laser (*p* = 0.27). **b** Ho:YAG: myocardial perfusion up to 12 months. An increase in myocardial perfusion was determined between laser treatment and control groups, however this effect was not statistically significant using the holmium laser system (*p* = 0.06)

Ho:YAG lasers yielded similar results. When SPECT and PET scans were analyzed for the outcome measure ischemia at rest and stress, there was no statistically significant difference between TMR and MM or sham procedures (EE -0.12, 95% CI -0.48 to 0.23, *p* = 0.27, Fig. 6a) noted up to and including 12 months of follow up. Changes in myocardial perfusion at rest and stress, measured by SPECT and PET, showed improvements in the TMR and TMR/CABG groups as compared to MM, Sham or sole TMR therapy (EE 0.36, 95% CI -0.02 to 0.74, *p* = 0.06, Fig. 6b). However this effect was not statistically significant up to and including 12 months of patient follow-up. Of note this analysis included 30 porcine subjects receiving TMR therapy, which were reported to have statistically significant increases in myocardial perfusion following a 6-month trial using PET scanning.

Both lasers were subsequently compared to see if there was a statistical significant difference between their effect on ischemia and myocardial perfusion. For this analysis time points that were available for each measured outcome in both laser groups were compared. This included up to 12 months of patient follow-up. For the comparison of CO_2_ versus Ho:YaG lasers and their effect on ischemia, 2 studies with a total 288 patients were compared with 3 studies of 347 participants for CO_2_ and Ho:YAG lasers, respectively. After including time points up to 12 months of patient follow-up there was no statistical significant difference noted between laser types on ischemia (*p* = 0.10). In comparing the effect of laser type on myocardial perfusion, 4 studies with 272 participants and 7 studies with 374 subjects using CO_2_ and Ho:YAG laser systems, respectively were compared. All time points available for both laser types for this outcome were included, up to 12 months of follow-up. Results showed that there is no statistical significance on the effect of myocardial perfusion between the two laser types (*p* = 0.58).

### Part II

#### Clinical outcomes

**Fig. 7:**
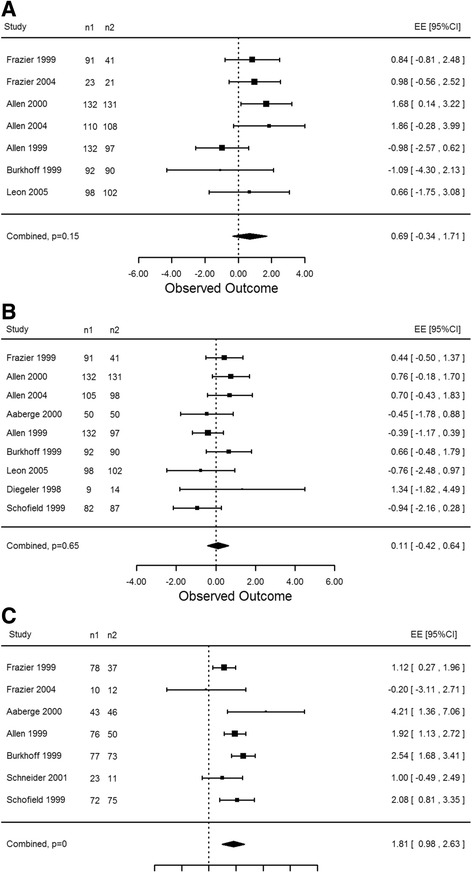
**a** Ho:YAG and CO_2_: 30 day survival. There was an increase in the survival rate of patients in the treatment group as compared to the control group (*p* = 0.15). **b** Ho:YAG and CO_2_: 12 month survival. Patients in the laser treatment group had a higher rate of survival as compared to those in the control group (*p* = 0.65). **c** Ho:YAG and CO_2_: angina reduction at 12 months. A statistical significance in angina reduction of two or more CCS classes was determined between the laser therapy group and the control group (*p* = 0.002)

Secondary endpoints for this meta-analysis determined the effect of laser therapy on three major clinical outcomes: survival, hospital re-admission and angina reduction. All subsequent analyses include results from both CO_2_ and Ho:YAG lasers combined at varying time points defined by eligible studies. Seven studies were used to determine the effect of TMR versus MM, sham or CABG patients on 30-day survival. Although results favoured laser therapy, the effect was not statistically significant (EE 0.69, 95% CI -0.34 to 1.71, *p* = 0.15, Fig. 7a). Similarly, nine studies were used to determine the effect of laser therapy on long-term survival rates, up to 12 months post treatment. A total of 1501 patients were considered in this analysis. Results indicate that although the treatment groups have higher survival rates there is no statistical significance difference between TMR or TMR/CABG and controls (EE 0.11, 95% CI -0.42 to 0.64, *p* = 0.65, Fig. 7b).

Further analysis examined hospital re-admission rates for patients undergoing laser therapy compared to MM. Three studies provided data for re-admissions at 12 months of follow-up. Again laser therapy was favoured, however this result was not considered significant (EE 2.71, 95% CI -1.89 to 7.31, *p* = 0.13). Seven studies reported data on angina class reduction of two or more classes. Three different time points were analyzed: 3 months, 6 months and 12 months post treatment. All three time frames showed statistically significant improvements in angina for the laser therapy group compared to control patients (EE 2.84, 95% CI 1.57 to 4.11, *p* = 0.006; EE 1.63, 95% CI 0.23 to 3.03, *p* = 0.03; EE 1.81, 95% CI 0.98 to 2.63, *p* = 0.002, respectively).

## Discussion

Multiple clinical trials have reported the benefits of laser therapy on patients with severe and diffuse coronary artery disease. Significant improvements in clinical symptoms have been demonstrated by many randomized clinical trials and evidence supporting reduced angina, inotropic support, ICU admissions, hospital LOS, and arrhythmias, as well, increased QOL and exercise tolerance have been demonstrated [28, 29, 34–36]. However many studies have argued that there is a lack of evidence supporting more objective measures of cardiac function such as myocardial perfusion, ischemia and LVEF [17, 32, 37]. Therefore this meta-analysis aimed to examine current literature to determine the effect of laser therapy on these outcomes. Almeda et al. [17] and Tasse et al. [37] both reviewed four multicentre clinical trials [34, 36, 38, 39] and their findings on myocardial perfusion and LVEF [17, 37]. They reported that although Frazier et al. [34] demonstrated a 20% increase in myocardial perfusion, Allen et al. [38] showed no significant change when compared to medical management. Furthermore two other papers showed increases in perfusion with thallium scans [40, 41] while three others reported no change [34], [42, 43]. Together, these findings contribute conflicting evidence and inconsistency towards efforts to determine whether or not TMR promotes angiogenesis and/or increased myocardial perfusion. Early results from Frazier et al. showed interesting findings from 31 patients receiving laser therapy [44]. All patients were subject to PET, dobutamine echocardiography, ^201^TI- SPECT and MUGA scans at 3 and 6 months post treatment. Three-month follow-up via SPECT displayed no change in perfusion in lased and non-lased segments, however on PET scans the ratio of subendocardial to subepicardial (SEn/SEp) perfusion increased by 14% (*p* < 0.001). Furthermore, at 6 months SPECT scans showed no change in perfusion while PET scans demonstrated improvements in 36% of lased segments. Similar results were reported by Cooley et al. whereby PET results indicated significant SEn/SEp perfusion changes in lased patients (*p* < 0.0001) but SPECT scans did not (*p* > 0.05) [40]. Furthermore they reported an accuracy of 82% and sensitivity of 89% with PET imaging analysis.

Measuring subendocardial and subepicardial perfusion is considerably important in evaluating laser effects on perfusion. Since it has been understood that TMR channels occlude via thrombosis, it is the initial blood flow from the left ventricle to the myocardial vascular plexus that alleviates ischemia in a viable area of myocardium [46]. In the past it has been hypothesized that camerosinuisoidal connections formed with the ventricle can develop into arteriolar channels or vessels, supporting the theory of increased subendocardial perfusion [44]. In addition it has been thought that blood flow can redistribute from areas of adequate perfusion (epicardium) to areas of inadequate perfusion (endocardium) [36]. The current consensus is that mechanical, thermal and oxidative stress in the surrounding myocardial tissue can elicit responses such as VEGF upregulation [47] and angiogenesis [45]. In either case, it may be that PET scans have the sensitivity necessary to detect these changes in perfusion while SPECT and MUGA scans do not. Results from this meta-analysis determined that there were no significant differences in myocardial perfusion between control and treatment groups at 3 or 12 months using SPECT, PET or MUGA scans. However 6-month follow up did show a significant improvement in myocardial perfusion using PET imaging. This result highlights a number of important points. (1) Imaging modalities need to have the capability of measuring subendocardial perfusion in TMR patients in order to detect increased collateral blood flow in the radial direction as opposed to the transmural (2) due to the cost of PET scans there are limited clinical trials performed on lased patients (3) the 6 month PET results from this analysis were based on three porcine models and therefore must be taken with light consideration. Despite this fact, they do coincide with other reports in human trials [44] and porcine models [4, 45] showcasing that in order to validate PET as a standard for follow up in TMR patients, there needs to be more data published on its efficacy to warrant the cost-benefit of using this imaging modality and lastly (5) more clinical trials are necessary to prove whether the increase in subendocardial perfusion is temporary or has a lasting effect in patients.

When CO_2_ and Ho:YAG lasers were analyzed individually, no statistically significant differences were found between CO_2_ and Ho:YAG lasers in any of the analyzed metrics (ischemia and myocardial perfusion) except for LVEF. The CO_2_ laser system did not demonstrate an improvement in the LVEF of lased patients as compared to the MM group. This may be due to conflicting reports on the contractility and wall motion noticed in TMR patients via MRI and echocardiography [13, 48–50]. More importantly, both CO_2_ and Ho:YAG lasers demonstrated an improvement in myocardial perfusion, however this increase was not considered significant. This could be the result of the fact that many studies in this category included SPECT and MUGA imaging as opposed to the more sensitive PET scan, which has demonstrated superiority in measuring subendocardial perfusion. When both lasers were compared against each other to determine whether the type of laser had an effect on myocardial perfusion and ischemia, up to a 12 month follow up, no statistically significant difference was noted. Despite the difference in thermal damage [51], thermoacoustics [52], thermal dispersion [53], and fibrosis [48] described between CO_2_ and Ho:YAG lasers, it has been difficult to prove superiority of one laser type over the other. Studies which report increased vascular density [20] with the Ho:YAG laser also report increased fibrosis [48] which may conflict with myocardial contractility. Therefore it is the balance of fibrosis and angiogenesis that is important in determining perfusion measures in lased hearts.

In an effort to answer whether TMR therapy has an effect on objective cardiac measures this meta-analysis examined short and long term survival rates and hospital re-admissions. There were no statistically significant differences between control and treatment groups in either 30-day or 12 month survival rates. However the estimated survival rate was higher for those receiving laser therapy as compared to control patients at both 30-days and 12 months. From the studies included in these subsections, 30 day mortality was attributed to unstable angina [34], sole CABG therapy [35], acute MI [39], LV dysfunction, ventricular fibrillation, respiratory insufficiency, multisystem organ failure [38] as well as higher preoperative patient risk scores from renal disease, IABP support, previous CABG, PTCA, HF, angina class and previous MI [54]. Long term mortality was associated with low LVEF, acute MI [34], sudden cardiac death of unknown etiology [30], cancer, intracerebral hemorrhage [13] as well as preoperative risk factors of age, EF, DM and dialysis [55]. It is possible that the statistically non-significant differences in survival rates associated with laser therapy could be a result of the significantly higher proportion of individuals with unstable angina reported at baseline in our analysis. In addition, many authors also argue that patients with severe symptoms are categorized into the treatment group in nonrandomized clinical trials. Studies that report higher than average survival rates claim that it is due to their stringent study selection criteria.

Survival and hospital re-admission rates were reportedly better for TMR patients than control, however this result was not significant. This is encouraging since many patients are re-admitted due to unstable angina and our preliminary baseline characteristics demonstrated a higher proportion of unstable angina in treatment patients. Angina reduction was also analyzed at 3, 6 and 12 months for both laser types collectively. All reported data included a reduction of at least two or more angina classifications. Patients in the treatment group experienced a statistically significant reduction in angina compared to control patients at all three time points. This is consistent with published literature demonstrating the known effect of TMR on angina relief, which can persist 5 years following laser therapy [56].

### Limitations

There were many challenges in putting together a comprehensive analysis on myocardial perfusion and imaging techniques. The primary difficulty arose in being able to standardize outcome measures from all studies. Due to the variability in endpoints and terminology used by each imaging modality, certain outcomes were combined in order to create categories for analysis. This paper only included 16 studies, which specifically looked at cardiac function via 3 imaging modalities, therefore the effect of MRI and ECHO was not taken into consideration. Furthermore, only data from control versus treatment groups were included and 3 out of 16 studies used a porcine model, due to low search results from PET imaging. Some analyses were confounded by time and laser type as multiple time points or laser types were included. All of these factors could have an influence on the results of this paper. In the future, pre versus post treatment imaging data, would need to be analyzed and combined with the current data to determine the overall effect of TMR. In addition MRI and ECHO studies could be included to provide further insight since MRI has been reported to show regional myocardial function with advanced spatial resolution [57].

## Conclusion

Currently, angiogenesis has been proposed as the leading theory behind TMR laser therapy, with reports of increased vascular density and neovascularization in lased myocardium. Despite the growing consensus for this theory it has been difficult to detect and monitor changes in perfusion. Nuclear imaging has emerged as a diagnostic tool used to identify perfusion defects, ischemic zones and blood flow in lased segments. Therefore it has become valuable in detecting perfusion changes. This meta-analysis has highlighted the importance of monitoring subendocardial perfusion in TMR patients as opposed to transmural or epicardial. This level of sensitivity can be achieved via nuclear PET imaging. Therefore the current debate regarding increased and/or decreased myocardial perfusion in TMR patients, may be a result of inconsistencies in the type of nuclear imaging modality being used. Future studies, which aim to analyze myocardial perfusion, should include PET scans within their study scope in order to provide sensitive and accurate measures of endocardial perfusion. These results may then explain the significant decrease in angina relief noted at long-term follow-up as well as the increases in myocardial perfusion, survival rates and hospital re-admissions. Further analysis is required to confirm the advantages of PET and its cost-benefit reward for CAD patients.

## Abbreviations

CABG: Coronary artery bypass graft
CAD: Coronary artery disease
Ho:YAG: Holmium yttrium-aluminum garnet
MI: Myocardial infarction
MM: Medical management
MUGA: Multigated acquisition scan
PCI: Percutaneous coronary interventions
PET: Photon emission tomography
PTCA: Percutaneous transluminal coronary angioplasty
RCT: Randomized clinical trial
SEM: Standard error of the mean
SPECT: Single photon emission computed tomography
TMR: Transmyocardial revascularization
TTE: Transthoracic echocardiography
